# How Do Climatic Factors Directly Influence the Incidence and Risk of Meningococcal Meningitis Across the African Meningitis Belt? A Narrative Literature Review

**DOI:** 10.1029/2025GH001749

**Published:** 2026-08-29

**Authors:** Molly Cliff, Kanny Diallo, Richard B. Yapi, Axelle B. D. Tano, Sally Jahn, Caroline M. Wainwright, Caroline Trotter

**Affiliations:** ^1^ Department of Veterinary Medicine University of Cambridge Cambridge UK; ^2^ Centre Suisse de Recherches Scientifiques en Côte d’Ivoire Abidjan Côte d’Ivoire; ^3^ Centre d’Entomologie Médicale et Vétérinaire Université Alassane Ouattara Bouaké Côte d’Ivoire; ^4^ Université Félix Houphouët‐Boigny de Cocody Abidjan Côte d’Ivoire; ^5^ MRC Centre for Global Infectious Disease Analysis School of Public Health Imperial College London London UK; ^6^ School of Earth, Environment, and Sustainability University of Leeds Leeds UK

**Keywords:** meningococcal meningitis, climate, atmospheric dust, climate change, Africa

## Abstract

Globally, the highest incidence of meningococcal meningitis occurs within the African meningitis belt, spanning 26 countries across sub‐Saharan Africa. Meningococcal meningitis incidence is highly seasonal in this region specifically, with outbreaks mostly occurring during the dry season, characterized by low rainfall and atmospheric humidity, high temperature, and increased dust and wind speed. The strong seasonality of meningococcal outbreaks coincides with seasonal variation in climatic factors. This multicollinearity can make it difficult to identify environmental drivers of disease and the mechanisms by which they operate. This review aims to collate existing evidence to better clarify the mechanisms by which climatic variables influence meningococcal meningitis incidence. We examined the impact of dust, wind speed, temperature, rainfall, and land cover on meningococcal meningitis outbreaks. Within the literature, atmospheric dust and wind speed had the strongest statistical association with meningococcal outbreaks and demonstrated greater predictive probability than other climatic variables. However, several climatic factors have demonstrable influences on one another, reflected in the seasonality of meningococcal meningitis. Atmospheric dust can reduce precipitation levels in part through its radiative properties. Decreased rainfall and increasing temperatures can dry out soil, increasing its availability to be uplifted as dust. Alongside, this lower atmospheric humidity increases evaporative demand, leading to faster soil moisture loss and enhanced surface drying. We argue that rainfall, temperature, and land cover variability may act as part of a broader climatic mechanism, increasing atmospheric dust. This increases the incidence and risk of meningococcal meningitis.

## Plain Language Summary

The African meningitis belt, spanning 26 countries across sub‐Saharan Africa from Senegal to Ethiopia, has a high burden of meningococcal meningitis. Outbreaks of meningococcal meningitis are highly seasonal in this region, peaking in the October–April dry season before subsiding with the onset of the rains. Due to the seasonality of meningococcal meningitis incidence, there is significant overlap among the climatic variables associated with the disease. This can make it difficult to identify the key climate variables that drive disease and the mechanisms by which they operate. In this literature review, we aim to understand the mechanism by which climatic factors influence meningococcal meningitis incidence. This review looked at the influence of dust, wind speed, rainfall, temperature, and land cover on meningococcal meningitis incidence. The literature consistently highlighted a strong association between dust, wind speed, and outbreaks of meningococcal meningitis. However, rainfall, temperature, and land cover can also affect atmospheric dust levels. This demonstrates the role of several climatic variables in a mechanism that increases the risk of meningococcal meningitis outbreaks.

## Key Points

- The seasonality of meningococcal meningitis outbreaks across the African meningitis belt demonstrates collinearity among climatic variables
- Atmospheric dust brought west by the Harmattan winds increases the incidence of meningococcal meningitis across sub‐Saharan Africa
- Lower levels of rainfall, as well as higher temperatures and increased soil drying can elevate dust levels, forming a climatic mechanism that influences meningococcal risk

## 1 Introduction

Globally, meningococcal meningitis, caused by the bacterium *Neisseria meningitidis*, represents a significant burden of disease among children and young adults. In 2019, there were approximately 400,000 cases of meningococcal meningitis, resulting in 32,000 deaths (Shen et al., 2024). Whilst the meningococcus is primarily a commensal bacterium, with its sole reservoir being the human nasopharynx, invasive disease occurs when the bacteria enter the bloodstream (Asturias et al., 2022). Meningitis then develops when meningococcal bacteria cross the blood‐brain barrier, reaching the subarachnoid space and multiply, leading to inflammation of the meninges (Sáez‐Llorens & McCracken, 2003). Globally, case fatality rates for meningococcal meningitis (up to 10%) are lower than for pneumococcal meningitis (up to 51%), which is caused by the bacterium *Streptococcus pneumoniae*. The suspected case definition for both forms of bacterial meningitis is defined as “the onset of fever ≥38.5°C with neck stiffness, altered consciousness, or other meningeal signs,” with the causative pathogen only being determined through further laboratory investigation (Soeters et al., 2019). Whilst case fatality rates are lower, a significant proportion of survivors of meningococcal meningitis suffer from neurological sequelae (Lucas et al., 2016). These sequelae, including epilepsy, hearing loss, and cognitive impairment, can represent a significant long‐term burden (Lucas et al., 2016).

**Figure 1:**
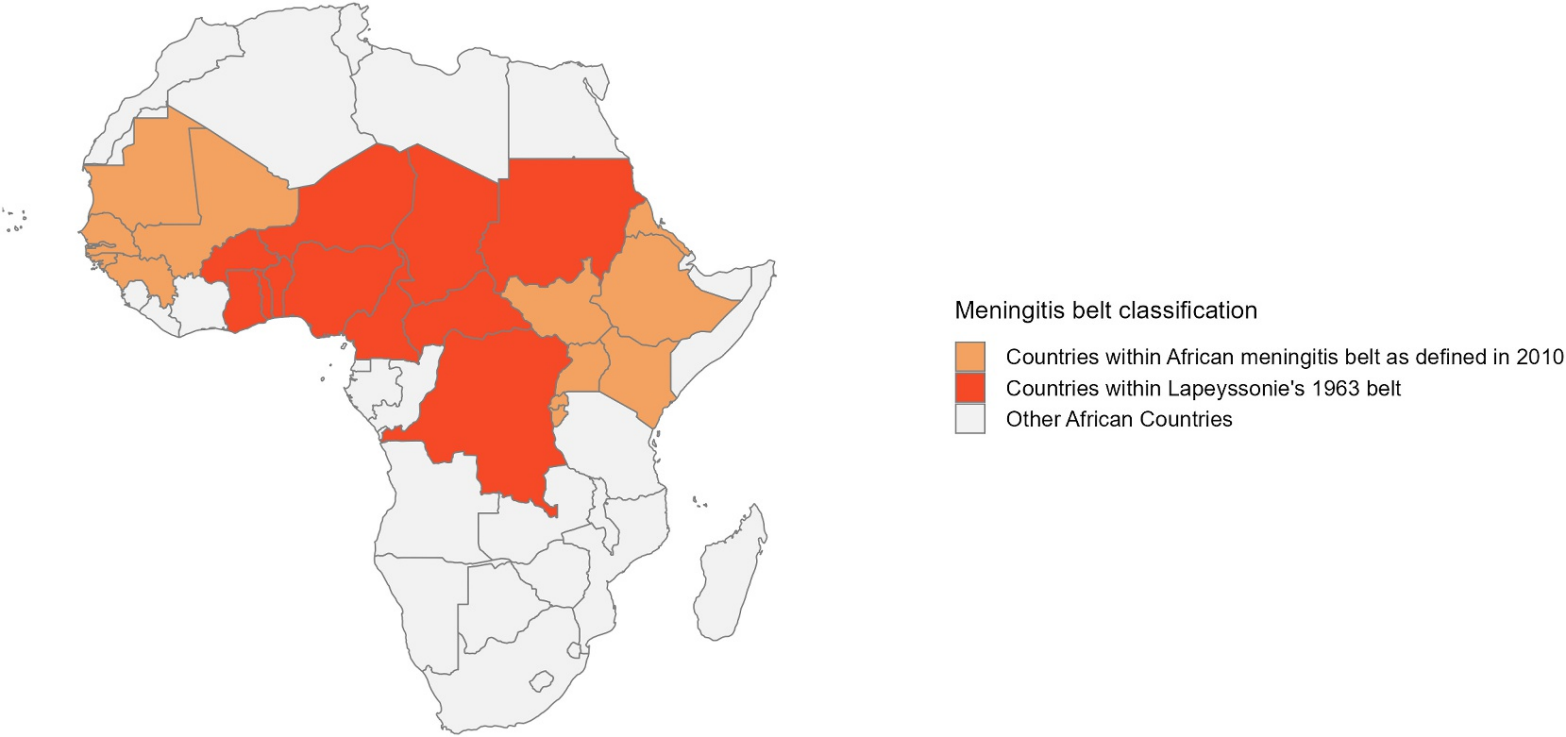
Map of Africa, highlighting the 26 countries of Africa's meningitis belt as defined in 2010 and the original 11 countries within Lapeyssonie's 1963 belt.

The highest incidence of meningococcal meningitis occurs within the meningitis belt in sub‐Saharan Africa, a region stretching from Senegal to Ethiopia (Mazamay et al., 2021). In 2019, globally, 50% of all meningitis cases in children under five occurred within the meningitis belt. Most of these cases would likely have been meningococcal meningitis (Wunrow et al., 2023). When first defined by Lapeyssonnie in 1963, the belt included 11 African countries, selected for having an average annual rainfall of 300–1,100 mm and a high incidence of meningococcal meningitis (Lapeyssonnie and Organisation mondiale de la Santé, 1963). With several additional assessments, the meningitis belt currently comprises 26 countries (Figure 1), having expanded to the east and west (Cheesbrough et al., 1995; Greenwood, 1987; Molesworth et al., 2002, 2003; Mpairwe and Matovu, 1971; Mustapha and Harrison, 2018). Meningococcal meningitis is highly seasonal, with cases peaking during the dry season (October–April) and declining with the onset of the African monsoon system (onset in June, within the Sahelian region of Africa) (Agier et al., 2013; Nicholson, 2013). Since the 1940s, epidemic cycles have occurred within the belt at irregular intervals, approximately every 8–12 years. From the 1980s onwards, the intervals between these epidemics have become shorter and more irregular (Mohammed et al., 2017). Irving et al. (2012) suggested that the periodicity of large‐scale epidemics was likely due to a combination of patterns in temporary immunity conferred by bacterial carriage alongside seasonal changes in bacterial transmission. Outside of Africa, meningococcal incidence has historically been low, with outbreaks peaking at approximately 14.0 cases per 100,000 population (Martin et al., 1998).

Following Lapeyssonnie's 1963 paper, several studies have highlighted a statistically significant relationship between meningococcal meningitis incidence and dust, wind and temperature. Across Africa, meningitis has been referred to as a disease of “wind and sun” in local languages, emphasizing the impact of climate on disease incidence (Besancenot et al., 1997; Colombini et al., 2009; Roberts, 2008). The significant association between meningococcal meningitis risk and climatic factors across the belt suggests that climate change has the potential to change future meningococcal disease dynamics.

However, there is considerable variation in the seasonality of meningococcal disease across geographic regions. In countries outside of Africa, meningococcal disease incidence peaks during the winter months (Collier, 1992; Lindsay et al., 2002). Several countries in both the Northern and Southern hemispheres have reported that bacterial meningitis incidence rises with colder temperatures. Such associations have been reported in several countries for example, Italy, China, Bangladesh, Cuba, the UK, New Zealand and Israel (Bai et al., 2017; Block et al., 1993; Bultó et al., 2006; Collier, 1992; Chowdhury et al., 2018; Lindsay et al., 2002; Michele et al., 2006). In countries in the Northern Hemisphere, meningococcal incidence increases between October and February, with a comparable increase occurring between June and October in the Southern Hemisphere (Paireau et al., 2016; van Kessel et al., 2019). Key variations in the regional seasonality of disease are indicative of the varying impact of climatic factors on meningococcal meningitis. A number of additional factors, including bacterial carriage rates, population immunity, and physical contact rates, all contribute to the incidence and risk of meningococcal meningitis. These factors are also able to be influenced by climatic conditions (Agier et al., 2017).

Although previous literature reviews have examined the impact of climatic factors on meningococcal meningitis incidence, our understanding of the influences of such factors has advanced in the last 10 years since the most recent publication (García‐Pando, Thomson, et al., 2014; Palmgren, 2009). Recent studies have placed increased emphasis on the role of human‐driven climate change in influencing meningococcal risk (Akanwake et al., 2022; Mazamay et al., 2020). This can impact both the timing and location of meningococcal outbreaks (Akanwake et al., 2022; Mazamay et al., 2020). Additionally, some studies have proposed the possibility of an optimal temperature for bacterial transmission (Mazamay et al., 2020; Oluwatimilehin et al., 2022). Studies have also examined the role of wind direction in influencing outbreak risk and have further highlighted the importance of the geographic position of countries within the meningitis belt (Nakazawa & Matsueda, 2017; Yarber et al., 2023). Overall, recent literature underscores an improved mechanistic understanding of the climatic influences on meningococcal outbreaks across the African meningitis belt.

This review examines climatic factors and their influence on the incidence and risk of meningococcal meningitis across the African meningitis belt, focusing on how climate change and variability can modify outbreak risk. Sub‐Saharan Africa is particularly vulnerable to climate change (Bedeke, 2023), which has the potential to influence the incidence and risk of meningococcal outbreaks (Mora et al., 2022). The Sahelian region of Africa is predicted to be a persistent climate change hotspot throughout the remainder of the 21st century (Diffenbaugh & Giorgi, 2012). Additionally, the strong seasonality of meningitis across the African meningitis belt demonstrates the considerable collinearity between climatic factors. This can make it difficult to disentangle the main drivers of disease. Within this review, we aim to collate existing evidence to better clarify the mechanisms by which climatic variables influence meningococcal meningitis incidence. Distinct from earlier reviews, this paper proposes a novel hypothesis identifying the key factors for increased meningococcal meningitis risk and incidence. This hypothesis emphasizes the central role of Saharan dust carried across West Africa by the Harmattan wind in increasing the risk of meningococcal meningitis outbreaks. Establishing climatic drivers for meningococcal meningitis alongside their mechanisms of impact is essential to understand the potential future burden of disease under a changing climate.

## 2 Methods

Within this review, we focused on how climatic factors that are sensitive to greenhouse gas (GHG) emissions may drive the risk of meningococcal meningitis outbreaks across the African meningitis belt. Rising GHG emissions trap atmospheric heat and increase the temperature of the Earth's surface (Filonchyk et al., 2024). Warming caused by GHGs can impact a variety of climatic mechanisms with significant impacts on human health. This includes increasing the occurrence of droughts and causing higher levels of particulate matter within dust storms, which could have direct impacts on meningococcal incidence (Mora et al., 2018).

We used a 2022 paper by Mora et al., which examined the impact of climatic hazards sensitive to greenhouse gas (GHG) emissions on human pathogenic disease, to guide the scope of our database searches (Mora et al., 2022). Climatic hazards sensitive to GHG emissions identified within Mora et al.'s paper were used as the basis for key search terms. By initially focusing on climatic hazards sensitive to GHG emissions, we were able to frame the literature review primarily within the context of climate change‐driven processes.

Due to the limited number of climatic hazards linked to meningitis identified in Mora et al.'s work, we broadened our selection to include additional climatic hazards with established associations with meningococcal meningitis from the wider literature (Table 1). We then translated the climatic hazards identified in Mora et al.'s work into climate variables. During this process, “Storms” as used in Mora et al. was separated into dust and wind speed, accounting for both dust storms and windstorms alongside the separate effects of these factors on meningococcal meningitis outbreaks. Rainstorms were covered by a separate rain variable. Climatic hazards considered included landcover change (deforestation and desertification), precipitation, temperature, wind speed, and dust. All climatic hazards identified within this review had a confirmed association with meningococcal meningitis outbreaks, as indicated in Table 1 (column‐ Association with meningococcal outbreaks).

**Table 1:** Search Topics, Corresponding Medical Subject Headings (MESH) and General Search Terms Used Within Our Narrative Literature Review

Climatic risk factor	Medical subject headings (MESH) terms used	Additional search terms used	Corresponding terms in Mora et al.	Association with meningococcal outbreaks
Landcover and landcover change (deforestation and desertification)	Climate	Land cover Deforestation Desertification	Natural cover change	Molesworth et al. (2002); Thomson et al. (2006)
Precipitation patterns	Rain Drought	Rainfall patterns Precipitation patterns	Precipitation and Floods	Thomson et al. (2006); Jackou‐Boulama et al. (2005)
Temperature	Temperature Hot temperature Climate	Heatwave	Drought and Heatwaves	Mazamay et al. (2020)
Wind speed	Wind Cyclonic storms Air	Windstorms Air movements	Storms	Sultan et al. (2005)
Dust	Dust atmosphere	Aerosol optical density Aerosol optical depth Dust concentration Dust storm	Storms	Molesworth et al. (2002); Thomson et al. (2006)

*Note*. Note that MESH headings are hierarchically organized terms used in PubMed to catalog and search relevant literature.

Although humidity has a known link to meningococcal outbreaks across the African meningitis belt, we did not include it as a search term, as it did not fall under any of the climatic hazards identified by Mora et al. (2022). Whilst atmospheric humidity has been considered in some studies, it was not included as a primary search variable, in part due to its collinearity, and overlap with rainfall, temperature, and dust‐related indicators. Furthermore, given the role of atmospheric dust transport in increasing the risk of meningococcal outbreaks, soil moisture may provide a more mechanistic representation of surface drying conditions that drive dust emissions. Nevertheless, due to its frequent appearance within the literature we included a dedicated discussion of the association of atmospheric humidity with meningococcal outbreaks.

Specific humidity represents the actual amount of water vapor present in the air and therefore reflects the true atmospheric moisture content. Absolute humidity also serves as a measure of water vapor present in the air but is expressed independently of air temperature. In contrast, relative humidity is temperature‐dependent, as it expresses the amount of water vapor in the air relative to the maximum amount the air can hold at a given temperature. Humidity is likely to be influenced by ongoing climate change, with different forms of humidity being variably affected.

There has been a global increase in specific humidity since the 1970s because of increasing temperatures, which improves atmospheric ability to hold water vapor (Gulev et al., 2023). It is likely that specific humidity will continue to increase in part due to rising sea surface temperatures, increasing levels of atmospheric moisture (Chadwick et al., 2016). Conversely, relative humidity has decreased globally since 2000, particularly in mid‐latitude regions of the Northern Hemisphere, where temperatures are increasing rapidly (Gulev et al., 2023). These changes are considered largely to be attributable to ongoing climate change (Willett et al., 2007).

Papers were considered if they had been published up to 2025, and we included papers published in English and French, given the large Francophone population within the meningitis belt. The scope of our narrative literature review included experimental, qualitative, and observational studies, as well as reviews and letters to the editor.

**Figure 2:**
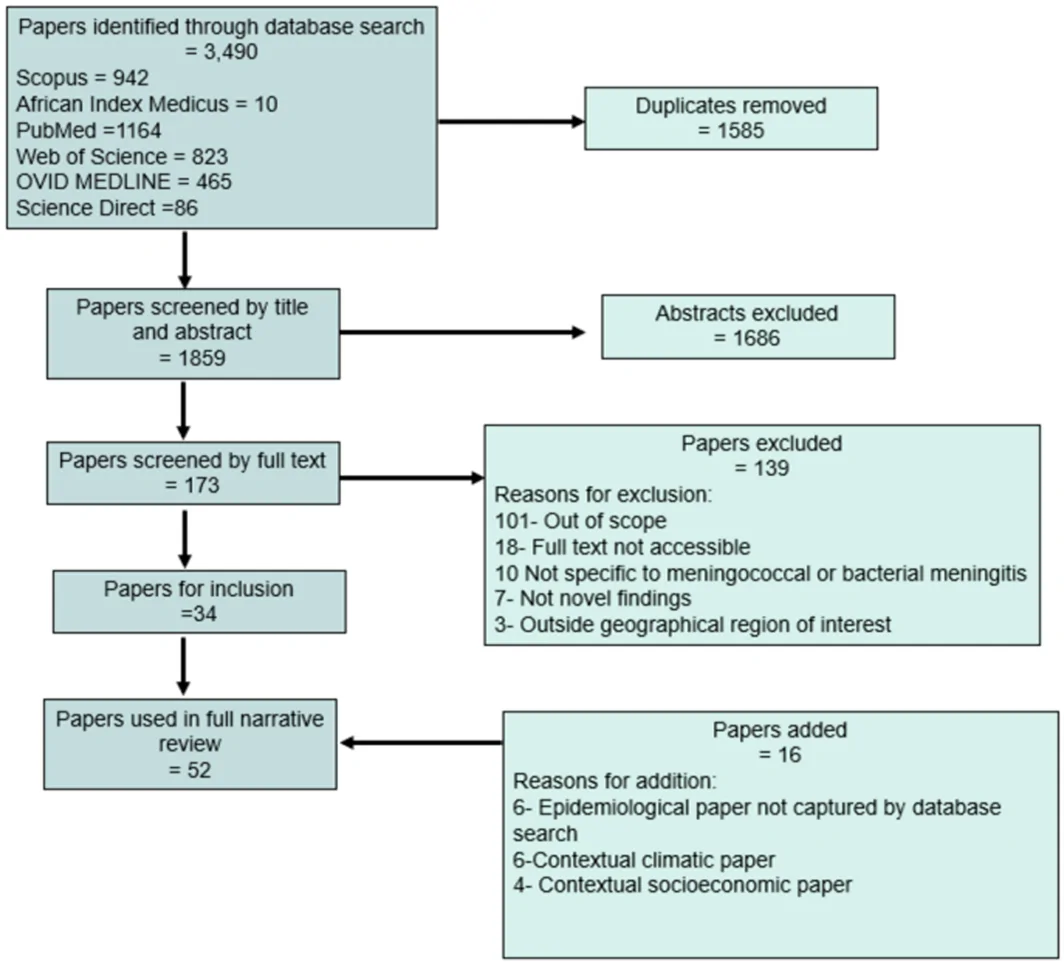
PRISMA chart of methodology used within narrative literature review.

The computerized search for this review included Medline terms and keywords relating to meningococcal meningitis and topics in Table 1. We used PubMed, Web of Science, ScienceDirect, Scopus, Medline, Global Health and Global Index Medicus to screen for appropriate abstracts to use within the review (Table 1). We searched for papers using both the MESH terms and additional search terms for each climatic risk factor, appending “meningococcal meningitis,” “pneumococcal meningitis,” “Haemophilus meningitis,” and “bacterial meningitis” to each query. We included all key causative agents of bacterial meningitis in our search strategies, as some published analyses use suspected case data that refer to meningitis as a general syndrome rather than specifying the causative pathogen. As the majority of bacterial meningitis outbreaks across the belt are caused by *Neisseria meningitidis*, we assumed that reports of suspected meningitis outbreaks would likely include meningococcal cases. Therefore, we included such reports within our literature review. Studies that explicitly stated that cases or outbreaks of bacterial meningitis were not due to meningococcus were excluded, allowing us to differentiate between meningococcal meningitis and suspected meningitis cases within our review. In the primary database search, we identified 1859 non‐duplicate texts, which were screened for scope and relevance based on their titles and abstracts. Following on from this, relevant articles were read in full, and references from included studies were reviewed for additional sources. Supporting climatic literature was also incorporated into the review to supplement the epidemiological evidence (Figure 2). We have included a table summarizing the results of statistical papers examining the relationship between meningococcal meningitis incidence and climatic variables within Table S1 of this paper.

## 3 Results

### 3.1 Climatic Seasonality of Meningococcal Meningitis

Meningococcal meningitis incidence across the African meningitis belt is highly seasonal (Greenwood, 1999) and is largely controlled by dust, wind direction and wind speed. These factors, alongside low rainfall, play a considerable role in predicting meningococcal meningitis outbreaks and influencing population movement (Lapeyssonnie and Organisation mondiale de la Santé, 1963). Disease incidence increases following the onset of the dry season (October–April), in line with the movement of the dry Harmattan winds from the Saharan desert (Palmgren, 2009) (Figures 3a–3c). Outbreaks of meningococcal meningitis then subside with the onset of the monsoonal rains, which begin in June and peak between July and September (Agier et al., 2013; Nicholson, 2013).

Figure 3(a) Seasonal cycle of district‐level bacterial meningitis cases across the African meningitis belt (2021–2025). Countries included: Angola, Benin, Burkina Faso, Burundi, Cameroon, Central African Republic, Chad, Côte d'Ivoire, Democratic Republic of the Congo, Ethiopia, Gambia, Ghana, Guinea, Guinea‐Bissau, Kenya, Mali, Mauritania, Niger, Nigeria, Senegal, Sierra Leone, South Sudan, Sudan, Tanzania, Togo and Uganda. Data from the Enhanced Surveillance of Meningitis in Africa Dashboard (World Health Organization Regional Office for Africa, 2025). (b) Historical record of district‐level bacterial meningitis cases across the African meningitis belt (2021–2025). Countries included: Angola, Benin, Burkina Faso, Burundi, Cameroon, Central African Republic, Chad, Côte d'Ivoire, Democratic Republic of the Congo, Ethiopia, Gambia, Ghana, Guinea, Guinea‐Bissau, Kenya, Mali, Mauritania, Niger, Nigeria, Senegal, Sierra Leone, South Sudan, Sudan, Tanzania, Togo and Uganda. Data from the Enhanced Surveillance of Meningitis in Africa Dashboard (World Health Organization Regional Office for Africa, 2025). (c) Seasonal cycle of climatic variables across the African meningitis belt (2003–2022). Here, the shaded ribbon represents the interquartile range, with the ribbon edges indicating the first and third quartiles across all countries. Data sources: wind speed, zonal and meridional wind and specific humidity, data from European Centre for Medium‐Range Weather Forecasts ERA5 reanalysis (Hersbach et al., 2023). Aerosol optical depth (dust) data from National Aeronautics and Space Administration MODIS (Levy & Hsu, 2015). Temperature data from National Aeronautics and Space Administration AIRS (GISTEMP Team, 2024; Lenssen et al., 2024). Rainfall data from Climate Hazards Group InfraRed Precipitation with Station data (CHIRPS) (Funk et al., 2015). Countries included: Angola, Benin, Burkina Faso, Burundi, Cameroon, Central African Republic, Chad, Côte d'Ivoire, Democratic Republic of the Congo, Ethiopia, Gambia, Ghana, Guinea, Guinea‐Bissau, Kenya, Mali, Mauritania, Niger, Nigeria, Senegal, Sierra Leone, South Sudan, Sudan, Tanzania, Togo and Uganda.

**Figure:**
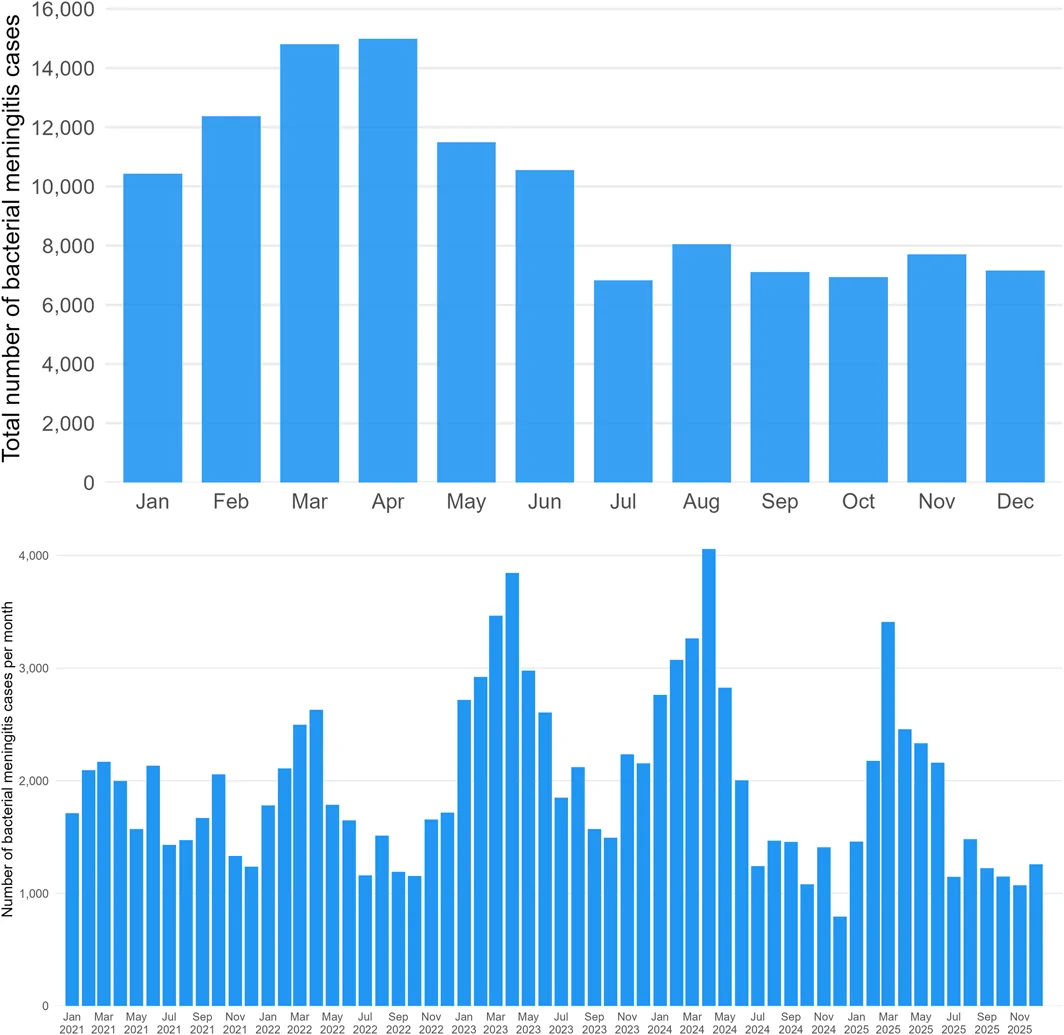

**Figure:**
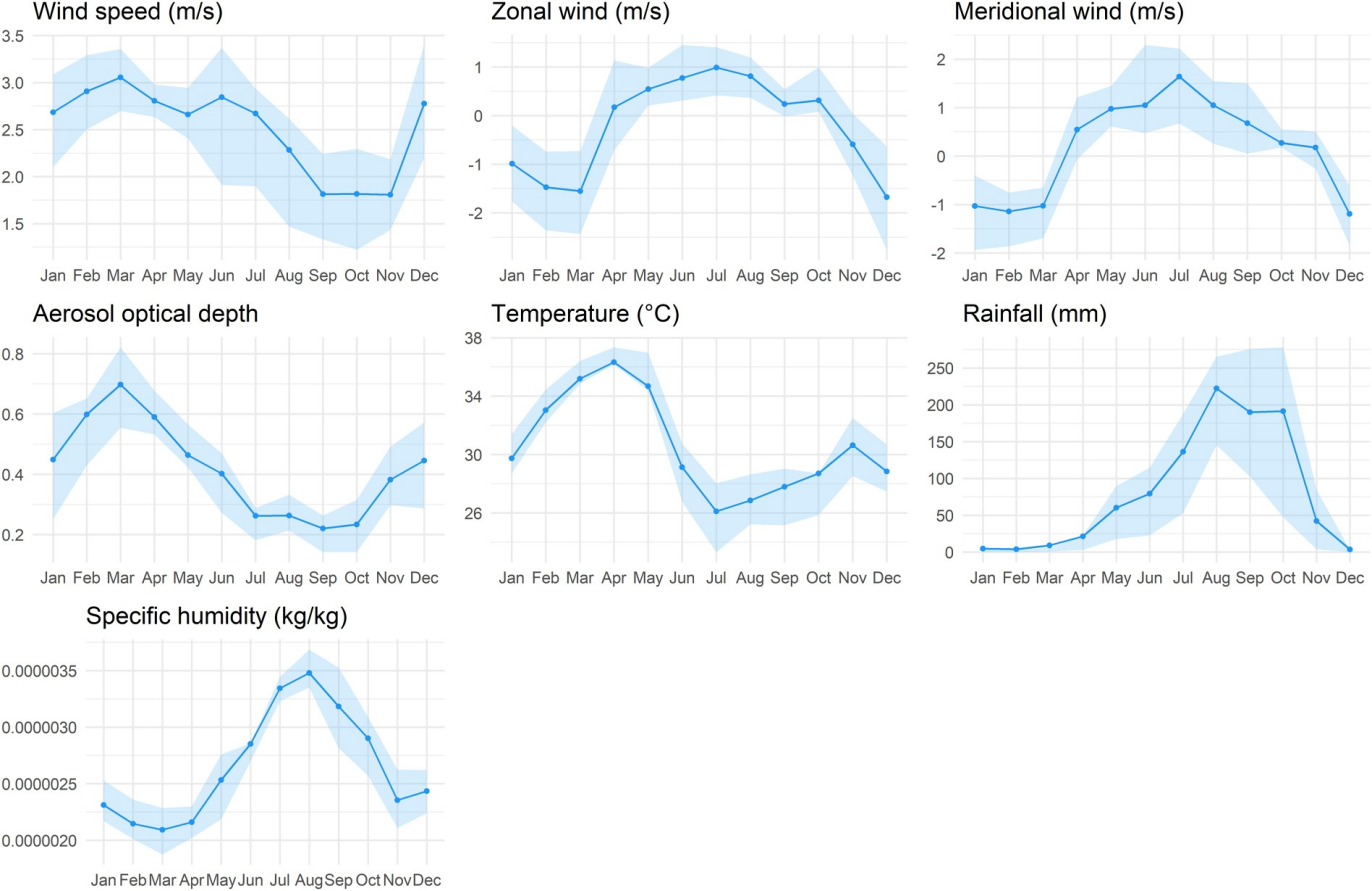

**Figure 4:**
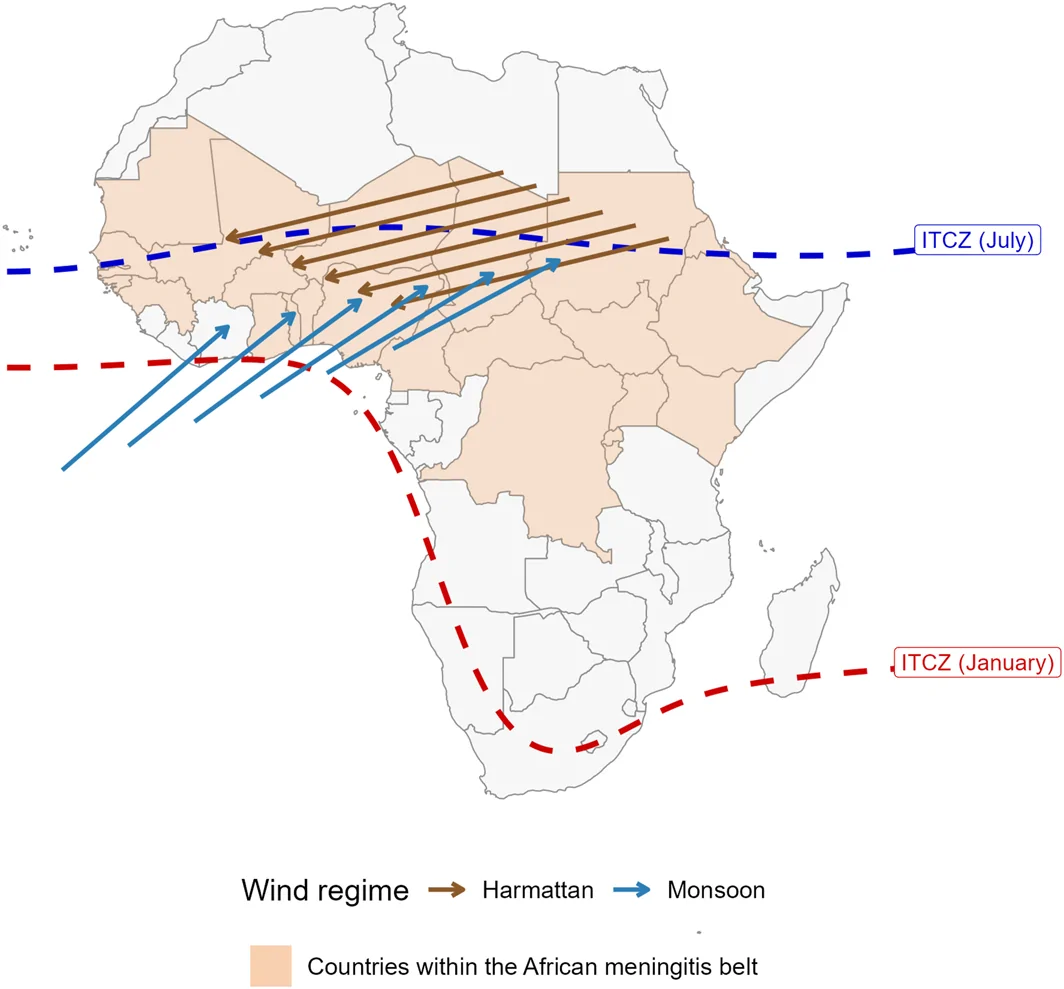
Map showing the position of the Intertropical Convergence Zone (ITCZ) at different times of year, as well as the direction of the associated monsoonal and Harmattan winds (Newton et al., 2006; Nguetsop et al., 2011).

The Harmattan winds lie within a group of trade winds which converge at the equator with the Intertropical Convergence Zone (ITCZ). The dynamic of the ITCZ and the associated Intertropical Front (ITF) (Lélé & Lamb, 2010; Mbourou et al., 1997; Nicholson, 2018) influence precipitation and dust patterns across the Sahel, creating distinct dry and rainy seasons. Across West Africa, the southern movement of the ITCZ aligns with the dry season, marked by the Harmattan winds. In contrast, the summer northern shift in the ITCZ is associated with monsoonal winds (Nicholson, 2018). As the ITCZ and ITF move northwards, southern monsoon winds from the Gulf of Guinea introduce moisture, increasing levels of rainfall (Figure 4).

During the dry season, dust localized within the lower levels of the atmosphere is carried largely from Bodele, Chad, west toward the Gulf of Guinea by the Harmattan winds. This leads to an increase in the absorbing aerosol index across the meningitis belt (Agier et al., 2013). This was assessed by Prospero et al., who used a Total Ozone Mapping Spectrometer (TOMS) to measure the global distribution of atmospheric dust sources. In this, the authors highlighted the Bodele Depression in Chad as the world's largest source of atmospheric dust (Prospero et al., 2002). Dust production in the Bodele Depression, occurs predominantly in October and April, in line with the seasonal movement of the Bodélé Low Level Jet winds (Goudie, 2014; Washington and Todd, 2005).

### 3.2 Statistical Associations

Across the climatic factors examined within our review, we found statistical associations between meningococcal meningitis epidemics and rainfall, temperature, landcover type, atmospheric dust and wind speed. However, there was considerable variation in the strength of such associations. Additionally, whilst not always explicitly included in studies, atmospheric humidity may influence meningococcal meningitis risk, but is often highly correlated with rainfall, temperature, and dust conditions. This suggests that the strength and independence of its predictive value for assessing meningitis may therefore be limited due to respective signals strongly captured by these other variables. Of all climatic factors studied, most evidence centered around the relationship between dust and meningococcal meningitis outbreaks driven by the Harmattan winds. This underscores the role of atmospheric dust carried by the Harmattan winds as a key driver in meningococcal outbreaks across the belt.

Greenwood et al. (1984) first identified a link between increased atmospheric dust and meningococcal meningitis outbreaks during a Nigerian meningitis outbreak, in which Harmattan winds lagged by 4 weeks correlated with increased hospital admissions. They noted that the association with lagged Harmattan winds was likely due to the intensity of the atmospheric dust from the Sahara. Only surface‐level and lower‐atmospheric measurements are important for assessing the health impacts of dust (Deroubaix et al., 2013). Following this, Besancenot et al. (1997) undertook a regression analysis within Benin and found that 14%–34.5% of temporal variability in disease outbreaks in the northern part of the country was a result of climatic factors, predominantly the Harmattan winds. This link could not be established in southern Benin, where the Harmattan has limited influence. Martiny and Chiapello examined the impact of dust on meningococcal meningitis incidence in West Africa using long‐term Sahelian aerosol data. The authors found that meningococcal outbreaks frequently occurred 0–2 weeks after an increase in atmospheric dust levels (Martiny and Chiapello, 2013). Many studies within the meningitis belt have concluded that dust and wind speed are directly associated with increased meningococcal meningitis incidence and outbreaks (Agier et al., 2013, 2017; Diokhane et al., 2016; García‐Pando, Stanton, et al., 2014; Nakazawa & Matsueda, 2017; Sultan et al., 2005). Dust forecasts have been effectively used to operationalize a meningitis early warning system with a 2‐week lead time across the African meningitis belt (Dione et al., 2022).

However, the spatial scale at which these analyses are carried out has a significant impact on statistical associations. Woringer et al. (2018) used a logistic regression model in Burkina Faso to analyse meningitis data at individual health centre catchment areas, focusing on small‐scale, microclimatic effects. When adjusting for calendar week as a model covariate, there was no statistically significant association between dust levels and meningitis cases. The authors attributed this lack of association to the larger, regional scale at which the Harmattan winds act. This can cause homogeneity in dust measurements across closely located health centre catchment areas. Similar results were observed in Paireau et al. (2014), which examined the correlation between wind‐blown dust and meningitis incidence at the health centre catchment area level. These studies highlight the difficulty of choosing the appropriate spatial scale for studying climate variability and meningitis (Pascual and Dobson, 2005). Additionally, through examining the relationship between atmospheric dust and meningitis incidence using national‐scale aggregated data, improvements could be made to forecasting approaches (García‐Pando, Stanton, et al., 2014; Yaka et al., 2008).

Several studies conducted across the African meningitis belt have found a statistical association between low rainfall and meningococcal incidence. In Niger, Jackou‐Boulama et al. (2005) found a statistically significant negative correlation between meningococcal meningitis and rainfall. Paireau et al. (2014) found that the occurrence of early rainfall in March was a protective factor against meningococcal epidemics in their own study in Niger. However, research has indicated that compared to factors such as dust and temperature, rainfall has a relatively weak association with meningococcal meningitis across the belt. Abdussalam et al. (2014) found that rainfall had a weak influence on meningitis incidence across dry season months in Northwest Nigeria (Abdussalam et al., 2014). Similar conclusions were reached in Ayanlande et al.’s paper (2020), which found that across Nigeria the relationship between precipitation and outbreaks was significantly weaker than that of dust and temperature. Whilst Molesworth et al. (2003) found an association between precipitation patterns and meningococcal meningitis within their logistic regression model, the authors suggested that this was likely linked with the strong statistical significance of dust. This is due to the increase in dust particles following the Sahelian droughts from the 1970s to the 1980s (Molesworth et al., 2003; Palmgren, 2009). The absence of rainfall may be associated with a driver of broader climatic conditions within the dry season, which have a more significant impact on disease incidence. Low rainfall may primarily function as a driver for increased atmospheric dust, which goes on to increase the incidence of meningococcal meningitis across the belt.

Across the belt, climatic relationships between meningococcal meningitis incidence and temperature remained varied. Several studies across the meningitis belt have found a statistically significant link between increasing temperatures and meningococcal meningitis incidence (Abdussalam et al., 2014; Akanwake et al., 2022; Dukic et al., 2012; Omoleke et al., 2018; Pinilla‐Monsalve et al., 2023). Adebayo (2001) conducted a 13‐year study in Yola State, Nigeria, and determined that 53% of the variation in meningitis incidence could be attributed to temperature changes. Research has also indicated that much of Central and Western Sub‐Saharan Africa is likely to continue to face a risk of meningitis epidemics due to increasing temperature variability (Chen et al., 2023). Additionally, Loh et al. (2013) were able to demonstrate that higher temperatures can further enhance meningococcal resistance to human immune responses. However, as with rainfall, some studies have found that dust and wind speed have stronger statistical associations with meningococcal incidence. Agier et al.'s (2013) study found stronger and more consistent correlations between dust, wind speed, and meningitis than between temperature and meningitis. Additionally, García‐Pando, Stanton, et al. (2014) compared a series of generalized linear models in their ability to predict meningitis incidence within Niger. Models utilizing zonal wind and dust as variables performed significantly better than those using temperature (García‐Pando, Stanton, et al., 2014). This further highlights the movement of dust carried by the Harmattan winds as a primary driver of disease.

Some studies have indicated the possibility of an optimal temperature for meningococcal transmission. Mazamay et al. (2020) found that, within the Democratic Republic of Congo, whilst maximum temperature had a negative correlation with meningitis incidence, mean temperature had a positive correlation with disease incidence. The authors highlighted that warmer regions within the DRC rarely have outbreaks of meningitis, suggesting that higher temperatures may inhibit bacterial growth, favoring autolysis. A similar result was observed by Oluwatimilehin et al. (2022), who found that minimum temperature was statistically positively associated with meningitis incidence. These studies are indicative of a possible non‐linear relationship between meningococcal meningitis incidence and temperature. Within their sub‐seasonal forecasting, Dione et al. (2022) suggested an operational threshold of air temperatures >30°C as favorable for meningitis outbreaks. During the dry season, higher temperatures may increase meningococcal incidence via the same mechanism that drives dust and wind. Whilst temperature may not act as a primary driver of meningococcal outbreaks, it may instead exacerbate risk factors such as dust, with a more central role in meningococcal transmission and invasion.

Across the Sahel, atmospheric humidity has a distinct seasonal pattern, increasing between May and October, before decreasing with the onset of the dry season (Martiny & Chiapello, 2013). Across the African meningitis belt several papers have found an association between humidity and meningitis. However, most of these papers have highlighted the cessation of meningitis because of increasing humidity (absolute and relative) rather than implicating low humidity in the occurrence of disease (Besancenot et al., 1997; Dukic et al., 2012; Paireau et al., 2014; Sultan et al., 2004). Notably, Pandya et al. (2015) found that an average of at least 40% relative humidity or greater for several weeks would naturally end a meningitis epidemic without the use of a reactive vaccination campaign. Additionally, several authors have suggested that the geographical boundaries of the meningitis belt are in part determined by higher humidity levels (Kelly‐Hope and Thomson, 2008; Sultan et al., 2004). It has been suggested that high relative humidity levels in Senegal are responsible for the country's lower burden of disease as compared to landlocked countries in the meningitis belt (Yarber et al., 2023). However, within these studies, there is limited discussion of the mechanistic association between atmospheric humidity and dust in driving meningococcal outbreaks. Much of the increase in disease is attributed to atmospheric humidity's role in exacerbating conditions suitable for bacterial invasion. Further studies should examine the potential association between increased humidity levels and reduced soil drying, which could lead to lower dust generation. Notably relative humidity has a key link to soil drying processes, given its direct relevance to evaporative demand and soil moisture loss.

As with temperature and rainfall, literature has suggested that humidity has a weaker association with meningococcal outbreaks as compared to atmospheric dust and windspeed. In their study across Burkina Faso, Nakazawa and Matsueda (2017), highlighted the notable contribution of zonal wind in driving the rate of change of the number of cases of meningococcal cases. A study by García‐Pando, Thomson, et al. (2014) and García‐Pando, Stanton, et al. (2014) similarly determined that when predicting seasonal incidence of bacterial meningitis across Niger, a model using zonal wind and atmospheric dust had the greatest predictive power. Within this study relative humidity prior to January did not have a significant association with seasonal meningococcal incidence at a country level underscoring the role of late season humidity in promoting the cessation of epidemics. Martiny and Chiapello (2013) examined the relationship between dust and absolute humidity across the meningitis season across Niger and Mali. They concluded that the timing of the meningitis season demonstrates the higher significance of dust in determining the onset of epidemics as opposed to humidity. Although disease incidence increases during the dry season, meningococcal cases predominately increase toward the end of the dry season, and are closely aligned with the first dust events of the year. In line with existing literature the authors highlight that the end of the meningitis season directly coincides with increasing humidity as a result of the arrival of the monsoonal season. This highlights that low levels of humidity alone are unable to drive meningococcal incidence, atmospheric dust and zonal wind serve as causative factors for outbreaks. However, increasing levels of humidity are sufficient at the end of the dry season to stop the meningitis season.

With outbreaks predominantly occurring within the Sahel and Sudanian regions of Africa, regional land coverage has a key role in predicting the location of meningococcal epidemics (Ayanlade et al., 2020; Molesworth et al., 2003). Molesworth et al.’s (2002) systematic review of district‐level meningitis outbreaks found that two‐thirds of all meningitis outbreaks occurred in countries in and surrounding the Sahel. In addition, their 2003 logistic regression model used land cover categories as part of their final model to predict district‐level outbreak occurrence (Molesworth et al., 2003). The authors found that regions without distinctive seasonal changes were less likely to experience epidemics than regions with pronounced wet and dry seasons (Molesworth et al., 2003; Thomson et al., 2006). Outbreaks of meningococcal meningitis rarely occur in forested or coastal regions, as high humidity reduces bacterial transmission (Kelly‐Hope and Thomson, 2008). Savory et al. (2006) conducted an evaluation of Molesworth's model using outbreak data from 2000 to 2004. The authors suggested that there had been a potential southward expansion of the meningitis belt, in part due to the Sahel's vulnerability to climate change and changing land‐use patterns (Savory et al., 2006). It was suggested that changes in land use, among other climatic factors, may have led to an increase in atmospheric dust. However, no studies have examined how changes in land cover and use can alter the risk of meningitis epidemics. With much of the world's land surface area representing a possible source of dust, this represents a considerable research gap.

### 3.3 Secondary Associations

Whilst considerable scientific literature has indicated that atmospheric dust, carried by Harmattan winds, increases population risk of meningococcal meningitis, the mechanisms behind this association remain unclear. It is thought that atmospheric dust carried by the Harmattan winds can lead to increased incidence and risk of meningococcal meningitis across the African meningitis belt by facilitating bacterial transmission and invasion. In Burkina Faso, Mueller found that suspected meningitis incidence was strongly associated with increased upper respiratory tract infection incidence but not with lower respiratory tract infection (Mueller, 2019). Mueller (2019) also suggested that lower levels of humidity, alongside high levels of atmospheric dust can increase levels of nasopharyngeal colonization through damage to the pharyngeal mucosa. In turn, this can lead to a higher number of asymptomatic *Neisseria meningitidis* carriers (Agier et al., 2013). Saharan dust has been demonstrated to exacerbate asthma and favor the establishment of respiratory tract infections (Jusot et al., 2017). Within their in vivo study examining pneumococcal meningitis, Jusot et al. (2017) highlighted that dust‐exposed phagocytes were functionally impaired. The authors suggested that dust‐induced disease susceptibility is likely due to the inhalation of dust causing an inflammatory lung condition, which, coupled with impaired phagocytic activity, allows for improved bacterial survival and increases the likelihood of crossing the blood‐brain barrier.

Although the climatic factors assessed within this review all have a demonstrable influence on meningococcal meningitis incidence, limitations in statistical modeling highlight the importance of indirect effects on pathogen transmission. This can be seen in a study by Thomson et al., which examined the impact of climatic anomalies on meningococcal incidence. In this study, climatic conditions at the start of the dry season were associated with outbreak occurrence several months later. However, given the short incubation period of meningococcal meningitis, weather conditions at the beginning of the dry season are unlikely to affect outbreaks occurring several months later (Thomson et al., 2006). Similar results were seen in Yarber et al.’s study (2023), examining the association between meningitis incidence and several dust indicators in Senegal. Here, the authors found that the onset of the meningitis season occurred approximately 10 weeks after dust levels began to rise. In these studies, the lagged impact of climatic variables on meningococcal incidence reflects the delayed association between climatic factors and meningitis incidence. In part, this may be due to the influence of indirect factors, including bacterial carriage rates, population immunity, and contact rates. In contrast, Dukić et al. (2012) highlighted the potential role of climatic factors as proxy variables for such indirect factors. This can be seen in that during the Harmattan season within the meningitis belt, overcrowding often occurs indoors as people try to protect themselves from the dusty winds and cold nights (Kaburi et al., 2017).

Climate change has exacerbated many of the risks associated with meningococcal meningitis outbreaks through increasing temperature variability, drought frequency, and extreme weather events (Tomalka et al., 2021). This can lead to greater resource scarcity and exacerbate population vulnerability. Climate change has also increased desertification across Africa (World Health Organization Regional Office for Africa, 2025), which could further increase the risk of meningococcal outbreaks through more frequent dust storms. Desertification also has an indirect effect on meningitis outbreaks through its link with population displacement, food and water shortages, and conflict (Goudie, 2019). Sahelian droughts between the 1970 and 80s caused widespread desertification, leading to swathes of previously fertile land used for farming becoming barren (Fensholt et al., 2017; Molesworth et al., 2003). Reductions in vegetation because of changes in land use alongside climate change can increase levels of atmospheric dust (Heald & Spracklen, 2015). Decreasing vegetation has also been shown to increase the occurrence of dust storms (Tegen et al., 2004). Furthermore, Ward et al. (2014) suggested that changes in land‐use contributed to an 18% increase in dust emissions over the past 200 years, with there likely being a further 5%–10% increase over the course of the 21st century. These significant changes in dust emissions are likely to have had an impact on population health and would likely influence the risk of meningococcal meningitis outbreaks.

Globally, from 2018 to 2022, as highlighted by Gebrechorkos et al. (2025) there has been a 74% expansion in areas affected by drought when compared with 1981–2017. This is largely thought to be a result of increasing atmospheric evaporative demand. Furthermore, in 2022 alone, 30% of the global land area was affected by moderate and extreme droughts (Gebrechorkos et al., 2025). Within regions of Africa which are highly affected by drought, such as East Africa (Gebrechorkos et al., 2025), there may be an escalating risk of meningococcal meningitis due to the worsening of climatic and societal pressures. Many countries within the African meningitis belt now report cases of bacterial meningitis across all months of the year (World Health Organisation Regional Office for Africa, 2025), a trend thought to be because of the changing climate (Akanwake et al., 2022).

## 4 Discussion

### 4.1 Climatic Mechanisms Influencing Meningococcal Meningitis

In this review, we have discussed how climatic factors may increase the risk and incidence of meningococcal meningitis across Africa. With the aim of this review being to develop a novel hypothesis identifying the key climatic factors responsible for increasing meningococcal meningitis risk across the African meningitis belt, we first evaluate existing epidemiological hypotheses. There are several hypotheses as to how climatic factors influence the risk of meningococcal meningitis epidemics. One hypothesis is that factors, such as temperature and humidity, could increase meningococcal survival and transmission via respiratory droplets during the dry season. This would lead to an increase in the number of carriers and, subsequently, the number of cases (Greenwood et al., 1984). In line with this, Ghipponi et al. found higher bacterial counts in the air during the dry season compared to the rainy season within their study across Mali (Ghipponi et al., 1971). Historically, evidence supporting this hypothesis has been limited. Trotter and Greenwood (2007) reviewed data from meningococcal carriage studies in the meningitis belt, and whilst there was significant variation in reported carriage rates, the study found no link to seasonality. Earlier studies included within the authors' review were likely limited by their shorter time scales and small sample sizes.

Recent studies have shown seasonal variation in carriage rates across Africa, with higher rates during the dry season. Although not explicitly noted in the associated publication, this first occurred in a Ghanaian longitudinal cohort study, where meningococcal carriage rates were substantially lower in November (4.4%) than in April (7.9%) (Cooper et al., 2019; Leimkugel et al., 2007). Several cross‐sectional carriage studies carried out across seven countries spanning the width of the African meningitis belt showed higher meningococcal carriage in the dry season (4.2%), as compared to the rainy season (3.1%) (The MenAfriCar Consortium et al., 2015). In a meta‐analysis including these, as well as other carriage studies, Cooper et al. (2019) found that meningococcal carriage odds increased during the dry season, then rose again during outbreaks in their mixed‐effects logistic regression model (Cooper et al., 2019).

An alternative hypothesis is that increased dust during the dry season increases bacterial invasion rates due to damage to the pharyngeal mucosa (Moore, 1992). Koutangni et al. evaluated this hypothesis by conducting a systematic review and meta‐analysis of monthly meningococcal meningitis incidence and carriage, using case‐carrier ratios across populations within the African meningitis belt. In this study, there was limited seasonal variation in meningococcal carriage rates. However, disease incidence was 15 times higher during the dry season, supporting the increased invasion hypothesis (Koutangni et al., 2015). The paper argues that differences in carriage rates are instead likely a result of long‐term variations in bacterial strain.

These hypotheses are combined in Mueller and Gessner's model, which suggests that the seasonal transition from the endemic rainy season to the hyperendemic dry season across the meningitis belt causes an increase in invasive disease amongst carriers. The authors additionally proposed that localized epidemics within the dry season are likely caused by increased meningococcal transmission (Mueller & Gessner, 2010). Irving et al.’s evaluation of this model concluded that seasonal changes in transmission play a greater role than meningococcal invasion when replicating observed disease epidemiology (Irving et al., 2012). However, whilst Irving et al.’s study examined large, country‐level epidemics, Koutangni et al. developed this model at a finer spatial scale, focusing on dry season hyperendemicity within Burkina Faso. This study determined that mathematical models required both increased transmission and invasion to reproduce observed hyperendemicity (Koutangni et al., 2019). Koutangni and Irving also suggested that immunity caused by disease and bacterial carriage may serve as an explanatory factor in understanding the epidemiology of bacterial meningitis across sub‐Saharan Africa.

The variety of mechanistic hypotheses regarding the impact of climate on influencing meningococcal disease and carriage is indicative of the complexity of interactions between climatic factors and bacterial dynamics. Although an association between climatic factors and meningococcal meningitis has been recognized since 1963, the underlying biological mechanisms remain poorly understood. In epidemiological research, there has been limited guidance on how climatic factors may influence dust concentrations, which, in turn, could impact meningococcal dynamics.

Amongst the climatic variables discussed within this paper, atmospheric dust has the most well‐established statistical association with meningococcal outbreaks across the belt. As we have suggested, several climatic factors may not directly affect meningococcal incidence but instead may act as a driver for increased atmospheric dust, which goes on to influence the risk of meningitis epidemics across Africa.

### 4.2 Mechanistic Links Between Dust, Land Cover, Precipitation, and Humidity

**Figure 5:**
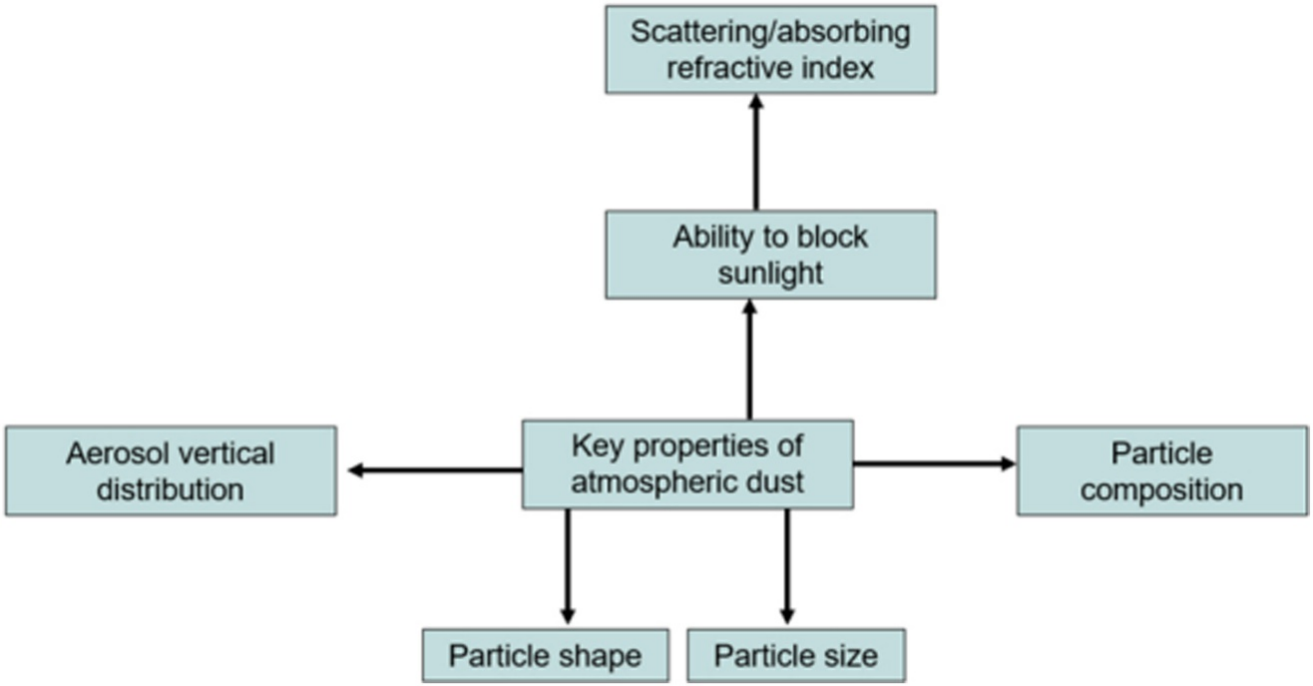
Characteristics of atmospheric dust which are informative within dust emission modeling.

As suggested by Molesworth et al. (2003), interactions between precipitation and dust across the Sahara and Sahel influence the risk of meningococcal outbreaks. Whilst it is thought that dust can drive precipitation levels across this region, published studies are discordant regarding the direction and magnitude of this impact (Evans et al., 2019). Although several studies have found that dust increases rainfall levels in the Sahel (Gu et al., 2012; Lau et al., 2009), most published literature instead suggests that dust decreases rainfall (Mahowald et al., 2010; Solmon et al., 2012; Yoshioka et al., 2007). This disparity is in part due to differences in model equations, as well as the importance of different variables and processes across both Global Climate Models (GCMs) and the Weather Research and Forecasting (WRF) model (Figure 5). GCMs are mathematical models, which aim to simulate the Earth's climate, through representing physical processes in the atmosphere, ocean and land. GCMs are based on a variety of climatic parameters and dynamics and can simulate both historical and future climatic conditions under several scenarios (Maraun, 2016). In contrast, the Weather Research and Forecast (WRF) is a regional community model, initially developed for mesoscale weather prediction (Powers et al., 2017). Whilst there is significant variation in the size and refractive index of dust across the Sahel, these are often assumed to be identical in climatic modeling (Adebiyi et al., 2023). The physical characteristics of dust have a key impact on dust albedo, leading to variation in its radiative properties. Additionally, some climatic models have demonstrated biases in dust emissions due to errors within surface wind fields (Evan, 2018).

The albedo of atmospheric dust has a key influence on Sahelian precipitation levels. When dust absorbs more radiation, it leads to warming of the lower atmosphere. This atmospheric warming is then able to destabilize the local atmosphere, providing increased energy for convection, ultimately strengthening monsoonal flow. The composition of dust, including its mineralogy and organic matter content, controls dust albedo. Variations in the composition and particle size of atmospheric dust can cause differing albedo (Figure 5). When atmospheric dust has a higher albedo and absorbs less light, the resulting reduction in atmospheric heating suppresses convection, weakening the West African monsoon system and decreasing Sahelian precipitation (Evans et al., 2019; Lau et al., 2009).

Atmospheric dust particles can also impact rainfall through their effect on cloud formation by acting as small cloud condensation nuclei (CCNs) (Chen et al., 2019). CCNs are a subset of atmospheric aerosols on which water vapor can condense. Whilst dust is a key source of CCNs, due to its small particle size, this leads to the formation of very small water droplets during condensation. This leads to the formation of clouds, composed of very small droplets, unable to merge into larger raindrops. This decreases rainfall levels across the Sahel (Rosenfeld et al., 2001).

Across the Sahel reduced precipitation levels can go onto increase levels of atmospheric dust, through drying our soil and decreasing regional vegetation coverage. This exposes more bare soil, which can be uplifted as dust during periods of high wind, further decreasing levels of rainfall (Marticorena & Bergametti, 1995; Rosenfeld et al., 2001). Alongside this, anthropogenic land use changes can increase topsoil exposure, which is vulnerable to wind erosion (Bergametti et al., 2017; Heald & Spracklen, 2015; Rosenfeld et al., 2001). Combined, these processes create a positive feedback loop whereby lower precipitation levels can increase levels of atmospheric dust, further suppressing rainfall levels across the Sahel. Humidity plays a critical role in driving precipitation, with atmospheric water vapor acting as the primary precursor to rainfall generation (Biasutti, 2019; Nicholson, 2013; Trenberth & Stepaniak, 2003). Future changes in rainfall across land will be driven in part by alterations in relative humidity and the movement of the convergence zones such as the ITCZ (Chadwick et al., 2014). This means that decreased relative humidity may lead to lower levels of rainfall and could potentially promote soil drying, improving conditions for uplift into atmospheric dust.

The impact of atmospheric dust on rainfall is complicated by surface cooling, as atmospheric dust's ability to reflect and absorb solar radiation can also heat the land below, increasing surface temperatures. When atmospheric dust blocks solar radiation from reaching the Earth's surface, this stabilizes the lower atmosphere, due to cooler surface temperatures. This then goes on to suppress convection and reduce precipitation levels (Evans et al., 2019). Across Africa, there is significant variation in surface cooling, with the effect being weaker over the reflective Sahara but stronger over the Sahel and West Africa, where there is lower surface albedo (Evans et al., 2019; Strong et al., 2015). As such, reduced atmospheric dust over the Sahel may increase levels of rainfall, due to its impact on surface cooling. Whilst this suggests that increased levels of atmospheric dust can suppress rainfall, this effect depends on the dust's ability to absorb and reflect light, as well as the underlying surface characteristics (Evans et al., 2019; Strong et al., 2015). This suggests that the influence of atmospheric dust on precipitation levels across Africa is largely region‐specific and dependent on dust type.

### 4.3 Mechanistic Links Between Temperature, Humidity, Dust, and Landcover

As temperatures increase, atmospheric evaporative demand also increases as the capacity of the atmosphere to hold water vapor is heightened. This can lead to discordant climatic effects due to moisture availability (Karimzadeh et al., 2025; Sheffield et al., 2012). When water is available, this can lead to increased evapotranspiration, increasing atmospheric moisture levels. This can contribute to an overall intensification of the hydrological cycle under global warming, alongside an increase in extremes, including both heavy rainfall events and drought conditions (Huntington, 2006; Trenberth, 1998). However, in moisture‐limited areas more likely to be affected by drought, rising temperatures can reduce relative humidity because they increase faster than atmospheric water content (Gebrechorkos et al., 2025; Sherwood & Fu, 2014). This can increase evaporative demand and contributes to decreased levels of soil moisture and vegetation stress.

Due to increasing temperatures' impact on evapotranspiration in part through humidity, there is a notable indirect effect on land cover. Ni et al. found that the water content of vegetated soil was 50% lower in summer than in autumn, due to increased evapotranspiration (Ni et al., 2019). Across Africa, high temperatures and drought are associated with lower soil moisture (Yuan et al., 2022). In hotter, drier seasons, the soil absorbs more energy, increasing soil temperatures and contributing to higher surface air temperatures (García‐García et al., 2023). This creates a feedback loop in which warmer air increases atmospheric water demand, driving further evaporation. This increases soil warming and drying, thereby impacting soil erodibility (Fryrear, 1995; Ozer, 2002). Increasing temperatures could also contribute to this feedback loop through drying soil, which can then be uplifted into the atmosphere during periods of increased wind speed (Figure 4). Uplift most often occurs in regions with sparse vegetation and in areas where soil is affected by land‐use changes (Miller & Tegen, 1998). Although this suggests that higher temperatures could go on to increase dust levels, further research is required to understand how this may vary across different regions with varying types of land and soil characteristics. Additionally, there has been increasing literature regarding the importance of humidity in impacting atmospheric dust levels due to its effect on the threshold friction velocity at which wind erosion is initiated (Csavina et al., 2014). Lower humidity levels usually favor drier soils that tend to favor dust emission due to reduced surface moisture and weaker particle binding. However, under specific low soil moisture conditions and depending on soil properties, particles may become more strongly bound, thereby limiting dust mobilization. High humidity leads to increased cohesion between dust particles, thus stronger winds are necessary to generate dust, leading to lower dust emission rates (Csavina et al., 2014).

The relationship between land cover and temperature is bidirectional. Vegetation types have a significant impact on land surface temperatures due to their impact on hydro‐climatic processes (Tesfaye et al., 2026). Positive anomalies in green vegetation across the Sahel after the monsoonal season have been demonstrated to increase evapotranspiration, offsetting potential temperature increases (Yu et al., 2017). A study by Tesfaye et al. (2026) has shown that, across the Nile River basin, improved vegetation density causes a cooling effect through increased evapotranspiration. Across the study area, this effect caused a temperature reduction across 43% of vegetated land, with vegetation change contributing to a net basin cooling of 0.02 ± 0.006°C per decade (Tesafye et al., 2026). However, a study examining the African Great Green Wall initiative found that whilst scenarios with higher vegetation coverage across the Sahel would increase precipitation levels, leading to decreased drought length, this was accompanied by higher levels of heat in the pre‐monsoonal season (Ingrosso & Pausata, 2024). Furthermore, whilst increasing green vegetation could create a feedback loop where increased evapotranspiration offsets potential temperature and further increases green vegetation, alterations in land‐use, often due to climate change can negate this (Brandt et al., 2017; Stith et al., 2016). Within Africa, whilst changes in precipitation have been variable, soil moisture is declining across the continent as a whole (Yuan et al., 2022). Soil drying driven by this mechanism, alongside reduced atmospheric humidity, could lead to higher levels of atmospheric dust, increasing the risk of meningococcal meningitis outbreaks.

Much of the Sahelian region of Africa is used for mixed farming, which can lead to alterations in land coverage at certain times of year (Bergametti et al., 2017). It has been suggested that anthropogenic land use changes from factors such as deforestation and agricultural expansion, compounded by climate change, could lead to increased levels of atmospheric dust through increasing levels of soil erosion (Evan et al., 2016; Papatheodorou & Achilleos, 2025; Telo da Gama, 2023). This could further contribute to increased incidence and risk of meningococcal meningitis across the African meningitis belt. Additionally, increased soil drying caused by drought or land‐cover change has caused several social, economic, and environmental issues across Africa, causing widespread population displacement. In both Ethiopia and Somalia, prolonged drought events coupled with a reduction in soil moisture have led to localized conflicts (Delbiso et al., 2017; Yuen et al., 2022). Such conflicts can lead to population displacement, in turn, increasing the risk of meningococcal transmission (IDMC, 2020).

### 4.4 Mechanistic Hypothesis

**Figure 6:**
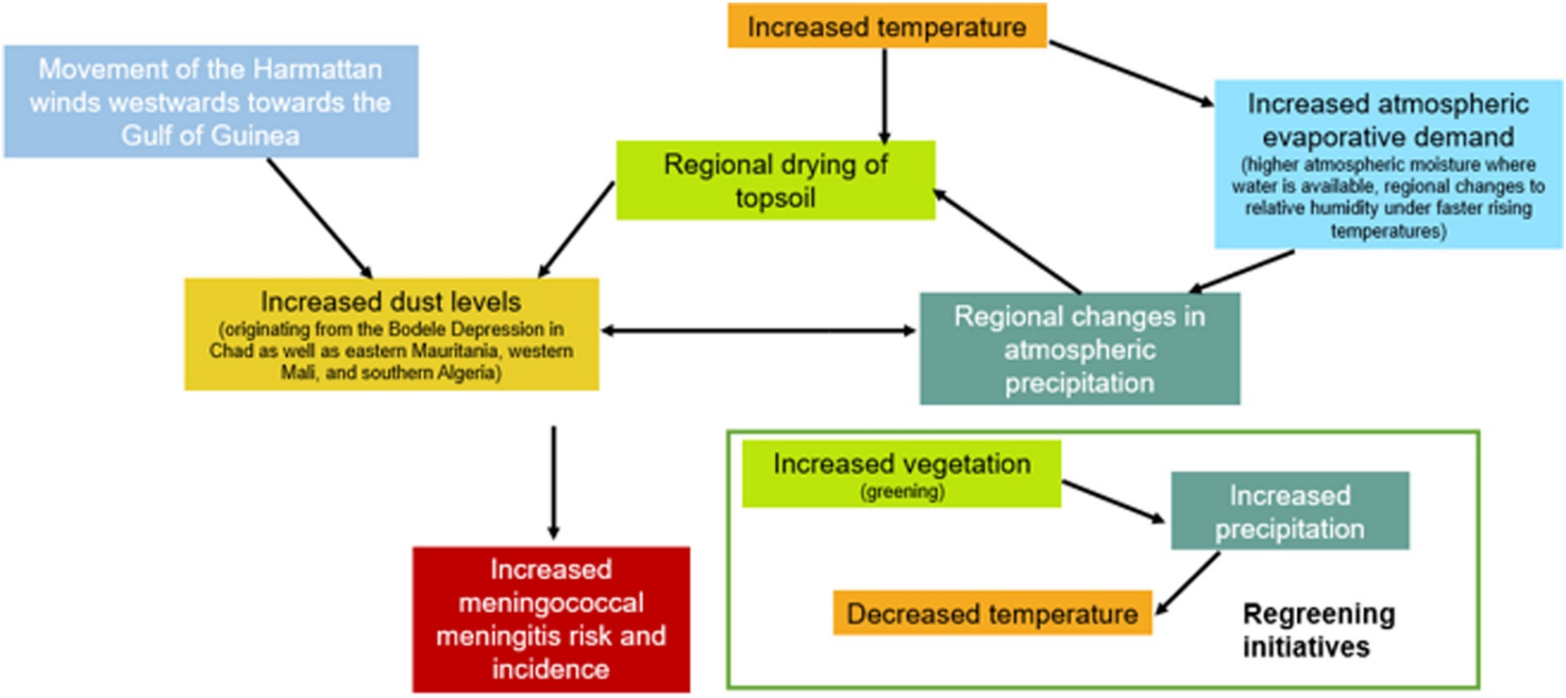
Hypothesized mechanistic pathway linking climatic factors to meningococcal meningitis risk.

We propose a mechanistic hypothesis for climatic effects on meningococcal outbreaks (Figure 6), combining the discussed evidence from both epidemiological and climate science papers. Initially, Saharan dust is carried largely from Bodele, Chad, west toward the Gulf of Guinea by the Harmattan winds. A region covering eastern Mauritania, western Mali and southern Algeria, also serves as a significant source of dust within sub‐Saharan Africa (Middleton & Goudie, 2001). Localized increases in atmospheric dust can influence the incidence of meningococcal meningitis due to seasonal increases in bacterial transmission and invasion.

Alongside this, both the radiative effects of atmospheric dust as well as its ability to act as cloud condensation nuclei can modulate local climates and decrease rainfall levels across the Sahel. Lower levels of rainfall can increase levels of soil drying, exposing more bare soil, which can be uplifted as dust during periods of high wind. This creates a positive feedback loop in which atmospheric dust can suppress rainfall and lead to improved conditions for dust emission. Increased land surface temperature can amplify this effect by reducing soil moisture levels and further decreasing vegetation levels. Finally, at higher temperatures, increased atmospheric evaporative demand can lead to varying environmental impacts, depending on moisture availability. In regions more likely to be impacted by drought, higher evaporative demand could further increase soil moisture loss and contribute to improved dust emission conditions.

This effect is partially mitigated by the effect of increased vegetation coverage in increasing evapotranspiration, promoting rainfall and reducing surface temperatures. However, ongoing climate change, compounded by anthropogenic changes, including deforestation and desertification, is likely to diminish vegetation coverage (Evan et al., 2016; Papatheodorou & Achilleos, 2025; Telo da Gama, 2023). This increases levels of soil drying, weakening this mitigation effect and potentially amplifying dust emissions.

Overall, this mechanistic hypothesis provides a framework to identify the key climatic drivers of meningococcal meningitis risk and incidence, highlighting the interactions between climatic factors. It emphasizes the central role of atmospheric dust movement across the Sahel in shaping meningococcal outbreak risk, highlighting the importance of the physical characteristics of dust in determining the strength of climatic and epidemiological impacts.

### 4.5 Atmospheric Dust Modeling and Future Projections of Precipitation

Due to the distinct role that dust has in controlling the risk of meningococcal meningitis outbreaks across the belt, dust emission models represent a key tool in understanding the climatic processes underlying disease risk. Dust emission models have been developed over several decades and have advanced our understanding of dust movement and the aeolian processes driving emission and deposition (Hennen et al., 2024). However, differences in assumptions about factors including particle size and composition can lead to substantial discrepancies in modeled dust emissions (Lunt & Valdes, 2002).

This variability is evident in several evaluations of dust aerosol modeling within the Coupled Model Intercomparison Project (CMIP6), which compare model outputs against reanalysis products and observational data sets. Zhao et al. found, in their global evaluation of 16 dust models, that variation in total dust emission levels between models was largely driven by differences in modeled surface winds (Zhao et al., 2022). Furthermore, a study by Kok et al. found that current climate models were unable to replicate long‐term increases in dust levels, biasing climate change projections (Kok et al., 2023). Whilst observational data can be used to bias correct errors in model projections, this suggests that it is difficult to accurately predict future trends in atmospheric dust. As atmospheric dust is a key driver of meningococcal outbreak risk across the belt, this has a key impact on the ability to predict future disease risk. With models showing discrepancies in their ability to predict future dust dynamics as well as a poor ability to replicate current variations in atmospheric dust, this highlights considerable uncertainty as to how meningococcal outbreak risk may shift under a changing climate.

With atmospheric dust having a notable impact on rainfall, as described within our mechanistic hypothesis, the magnitude and direction of projected changes in precipitation levels can vary significantly across the Sahel (Dosio et al., 2020; Tomalka et al., 2021). Although dust albedo has a key impact on climatic processes, there is little certainty in the amount of shortwave radiation absorbed by dust in the atmosphere (Li et al., 2021). Furthermore, whilst studies have suggested that the frequency of droughts is likely to increase in the future (Yahaya et al., 2024), potentially increasing levels of atmospheric dust, the overall direction and magnitude of precipitation changes vary across studies. Alongside atmospheric dust factors, changes in temperature across the North Atlantic and Mediterranean regions and uncertainty in anthropogenic aerosol radiative forcing have also been linked to uncertainty in future precipitation levels across models (Monerie, Biasutti, et al., 2023; Monerie, Dittus, et al., 2023; Monerie et al., 2020).

Across West Africa, where the highest risk of meningococcal meningitis lies (Cliff et al., 2026; Molesworth et al., 2003), whilst most climate models predict a statistically significant change in mean precipitation, there is debate over its direction and magnitude (Dosio et al., 2020). Over much of West and Central Africa less than 80% of models show agreement in total near‐term (2021–2040) precipitation levels across multiple climatic scenarios (Gutierrez et al., 2021) As such, there is limited consensus regarding the impact of climate change on both the geography of the meningitis belt and the intensity of epidemics within affected regions. This has key significance within public health resource planning as current constraints in global health funding necessitate the reprioritization of outbreak control resources. With limited supplies of meningococcal vaccine, anticipating future rainfall and dust patterns could enable prioritization of vaccination campaigns in areas where epidemics are more likely to persist (Meningitis Research Foundation, 2025; Pandya et al., 2015).

## 5 Conclusions

Climatic risk factors have a discernible and distinct influence on the global incidence and risk of meningococcal meningitis outbreaks. Of the climatic risk factors discussed within this literature review, dust and wind have the most substantial and well‐evidenced effect on meningococcal meningitis incidence, impacting disease risk both in and out of Africa. Multiple studies have underscored the relative statistical importance of these factors in predicting meningococcal meningitis outbreaks, compared to variables such as humidity, rainfall and temperature. Whilst rainfall, humidity, temperature and land cover may not act as primary drivers of meningococcal disease, they are likely to exacerbate risk factors that play a more significant role in disease transmission. Dust has a considerable influence on precipitation levels through its ability to act as cloud condensation nuclei and its radiative effects. Increasing temperatures are also able to exacerbate outbreak risk through soil drying, leading to the increased uplift of dust. Within the context of a changing climate, it is crucial to understand the differential interactions between climatic factors as well as their impact on meningococcal incidence and risk. Whilst there is uncertainty across climatic models regarding projections of dust, certain regions of the meningitis belt within West Africa may likely be at increasing risk of outbreaks because of more frequent droughts and higher levels of atmospheric dust.

## Conflict of Interest

The authors declare no conflicts of interest relevant to this study.

## Supporting information

Table S1

## Data Availability

All data used within this narrative literature review to create Figures 3a–3c are publicly available. Epidemiological data of district‐level bacterial meningitis cases across the African meningitis belt (2021–2025) in Figures 3a and 3b can be found at the Enhanced Surveillance of Meningitis in Africa Dashboard via: https://app.powerbi.com/view?r=eyJrIjoiMzExZTUzZDAtMmQ5Ni00YjkxLWFmZmItODljOWZhOTMyM2Y3IiwidCI6ImY2MTBjMGI3LWJkMjQtNGIzOS04MTBiLTNkYzI4MGFmYjU5MCIsImMiOjh9 with no access conditions. The specific humidity, wind speed, zonal wind speed, and meridional wind speed data used within Figure 3c are available from the European Centre for Medium‐Range Weather Forecasts (ECMWF) ERA5 reanalysis on the Copernicus Climate Data Store via https://doi.org/10.24381/cds.f17050d7 under the CC‐BY licence (Hersbach et al., 2023). The rainfall data used within Figure 3c are available from Climate Hazards Group InfraRed Precipitation with Station data (CHIRPS) via https://www.chc.ucsb.edu/data/chirps (Funk et al., 2015). CHIRPS data is in the public domain as registered with Creative Commons. The aerosol optical depth (dust) data used within Figure 3c are available from the National Aeronautics and Space Administration MODIS via http://dx.doi.org/10.5067/MODIS/MOD04_L2.061 (Levy & Hsu, 2015). This data set is openly shared, without restriction. The temperature data used within Figure 3c are available from the GISS Surface Temperature Analysis (GISTEMP), via https://data.giss.nasa.gov/gistemp/ (GISTEMP Team, 2024; Lenssen et al., 2024) and is publicly available.
