# Supplementary material for: How Do Climatic Factors Directly Influence the Incidence and Risk of Meningococcal Meningitis Across the African Meningitis Belt? A Narrative Literature Review
Source: Geohealth. 2026 Aug 29;10(9):e2025GH001749. doi: 10.1029/2025GH001749 (PMC13525485; doi:10.1029/2025GH001749)
Supplement: Supplementary file 1 [file GH2-10-e2025GH001749-s001.pdf]

GeoHealth

Supporting Information for

How Do Climatic Factors Directly Influence the Incidence and Risk of Meningococcal Meningitis Across the African Meningitis Belt? A Narrative Literature Review

Molly Cliff[1], Kanny Diallo[2], Richard Yapi[2,3], Devernode Tano[2,4], Sally Jahn[5], Caroline Wainwright[6], Caroline Trotter[1]

1. University of Cambridge Department of Veterinary Medicine, Cambridge, England, UK. 2. Centre Suisse de Recherches Scientifiques de Côte d'Ivoire, Abidjan,Côte d'Ivoire. 3. Centre d'Entomologie Médicale et Vétérinaire, Université Alassane Ouattara, Bouaké, Côte d'Ivoire.4. Université Félix Houphouët-Boigny de Cocody, Abidjan, Côte d'Ivoire 5. MRC Centre for Global Infectious Disease Analysis, School of Public Health, Imperial College London, London, United Kingdom. 6. School of Earth and Environment, University of Leeds, Leeds, UK. Corresponding author: Molly Cliff (mvc32@cam.ac.uk)

Contents of this file

Table S1

Introduction

Within the supporting information, we have included a table summarizing each paper included within our narrative literature review that specifically carried out a statistical investigation of meningococcal meningitis within the African meningitis belt. Within this table, we have outlined the country/region each study took place in, the climatic variables examined, and the study conclusions. Studies within this table were predominantly found within our database search as described in the methodology of the paper, with studies added in due to their epidemiological relevance. We did not include all studies examined within this literature review due to the heterogeneity in studies covered, including several climate and socioeconomic papers, in vivo studies and literature reviews. As this paper is a narrative literature review, no data processing steps were carried out.

Study	Year	Region	Climatic factors examined	Key conclusions
Lapeyssonnie and Organisation mondiale de la Santé	1963	African continent (no country focus)	Rainfall	Defined the boundaries of the African meningitis belt using rainfall (annual rainfall isohyets of 300 mm in the north, and 1,100 mm in the south)
Agier et al	2013	Niger	Temperature, relative humidity, and atmospheric dust	Dust has a stronger association with meningitis incidence as compared to other factors, with there being considerable similarity between districts in the evolution of the time-lags between the seasonal component of dust and meningitis.
Greenwood et al	1984	Nigeria	Temperature, absolute humidity, rainfall, and harmattan wind intensity	Meningitis outbreaks show a strong seasonal pattern in line with the movement of the Harmattan winds and cease at the onset of the monsoonal rains. Within Nigeria, the outbreaks of meningococcal meningitis had a positive correlation with maximum temperature and a weaker, but significant, negative correlation with absolute humidity and rainfall
Besancenot et al	1997	Benin	Temperature, relative humidity, atmospheric dust	14 to 34.5% of the temporal variability of meningitis disease was thought to be due to the movement of the Harmattan winds, as well as low humidity levels within Northern Benin. This association was not found within the south of the country, where the Harmattan winds have limited influence.
Martiny and Chiapello	2013	Niger and Mali	Atmospheric dust and absolute humidity	Meningococcal outbreaks frequently occur 0-2 weeks after an increase in atmospheric dust levels, with most outbreaks occurring between February and April. Low humidity is also associated with meningitis outbreaks
Diokhane et al	2016	Senegal	Atmospheric dust	Higher incidence of meningitis in Dakar, Senegal, aligned with increased atmospheric dust levels and particulate matter
García-Pando et al	2014	Niger	Zonal and meridional wind, wind speed, specific, absolute and relative humidity, temperature and atmospheric dust	Out of all variables, zonal wind had the strongest association with meningitis outbreaks and improved model fit the most. An additional model incorporating zonal wind and atmospheric dust levels also performed well
Nakazawa & Matsueda	2017	Burkina Faso	Windspeed, relative humidity, rainfall,	Dust and temperature are strongly associated with meningitis incidence, but disease incidence peaks several months after significant increases in

			temperature and atmospheric dust	northeasterly winds. Both wind direction and humidity can be used to predict outbreaks of meningitis
Sultan et al	2005	Mali	Windspeed, humidity (on pressure levels) , rainfall, temperature and atmospheric dust	Both the winter maximum of the Harmattan winds and the peak of meningitis incidence within Mali occur during the same week of the year and are highly correlated
Woringer et al	2018	Burkina Faso	Atmospheric dust	Whilst dust levels were associated with meningitis epidemics in initial analyses, when controlling for calendar week by including it as a model covariate, there was no statistically significant association between dust levels and meningitis cases.
Paireau et al	2014	Niger	Relative humidity, temperature, rainfall, meridional and zonal wind and atmospheric dust	Mean relative humidity from November to June and the occurrence of early rains in March were protective factors against meningococcal meningitis incidence. Spatial scaling of the study may have caused the limited association with atmospheric dust
Yaka et al	2008	Niger and Burkina Faso	Wind speed, surface temperature and specific and relative humidity	The Harmattan winds within Niger and Burkina Faso partly explain meningitis disease incidence. However, whilst statistical models based only on climatic variables show that 25% in Niger can be explained, such models are unable to replicate disease dynamics within Burkina Faso
Jackou-Boulama et al.	2005	Niger	Rainfall	Statistically significant negative correlation between meningococcal meningitis and rainfall
Abdussalam et al	2014	Nigeria	Temperature, rainfall, relative humidity, wind speed and atmospheric dust	Monthly mean values of daily temperature, humidity and atmospheric dust levels had the strongest association with meningococcal incidence
Ayanlande et al	2020	Sahelian region (no country focus)	Temperature, rainfall, atmospheric dust and landcover	Across the Sahelian region, there is a statistically significant relationship between meningitis incidence and both dust and temperature. The relationship between temperature and meningitis incidence is also present within the Sudanian region of Africa.
Molesworth et al	2003	African continent (no country focus)	Rainfall, absolute humidity, atmospheric dust and landcover	Absolute humidity, dust and rainfall seasonal categories, as well as landcover type, all had a statistical association with the presence/absence of meningitis outbreaks. Humidity and landcover type

				had the strongest association and were used within the final model
Akanwake et al	2022	Ghana	Temperature, relative and absolute humidity and atmospheric dust	Local communities have good knowledge of the impact of high temperature, low humidity and the Harmattan winds in increasing meningitis incidence
Dukic et al	2012	Ghana	Temperature, relative humidity, windspeed and atmospheric dust	Meteorological patterns can explain variations in meningitis incidence, particularly during large outbreaks. Maximum temperature and relative humidity had the strongest associations
Adebayo	2001	Nigeria	Temperature	53% of variations in meningitis incidence can be attributed to changes in temperature
Chen et al	2023	Global (discussion of sub-Saharan African)	Temperature	Sub-Saharan Africa remains as one of the most at risk regions globally for meningitis outbreaks due to increasing levels of temperature variability
Mazamay et al	2020	Democratic Republic of the Congo	Temperature and land cover	Increased incidence of meningitis found within in urban health zones than in rural conditions. Mean temperature but not maximum, is associated with meningitis incidence
Oluwatimilehin et al	2022	Nigeria	Rainfall and temperature	Positive associations were found between minimum temperature and meningitis, but not maximum temperature, with no association found with rainfall
Yarber et al	2023	Senegal	Atmospheric dust, temperature, relative humidity, wind speed, and wind direction	Onset of the meningitis season occurs approximately 10 weeks after dust levels began to rise. High humidity in Senegal may decrease incidence respective to landlocked belt countries
Thomson et al	2006	Niger, Mali and Togo	Landcover, atmospheric dust and rainfall	Anomalies in meningococcal epidemic occurrence were strongly associated with anomalies in dust and rainfall levels
Savory et al	2006	African continent (no country focus)	Rainfall, absolute humidity, atmospheric dust and landcover	Epidemics of meningococcal meningitis may be occurring south of the Sahel, consistent with regional climatic changes

Table S1. Descriptive table summarizing the statistical studies utilized within our narrative literature review examining the epidemiological links between climate and meningococcal meningitis within the African meningitis belt.
